# End-of-life decisions in Slovenian ICUs: a cross-sectional survey

**DOI:** 10.1186/cc12468

**Published:** 2013-03-19

**Authors:** S Grosek, M Orazem, M Kanic, G Vidmar, U Groselj

**Affiliations:** 1 University Medical Centre Ljubljana, Slovenia; 2 University of Ljubljana, Slovenia; 3 University Rehabilitation Institute, Republic of Slovenia, Ljubljana, Slovenia

## Introduction

The purpose of our study was to assess the attitudes of Slovenian intensivists towards end-of-life (EOL) decision-making and to analyze the decision-making process in their clinical practice.

## Methods

A cross-sectional survey among Slovenian intensivists and intensive care medicine residents from 35 different ICUs was performed using a questionnaire containing 43 questions about views on EOL decision-making. Fisher's exact test and the Fisher-Freeman-Halton test were applied to cross-tabulated data; significance level was set at *P *≤0.001 due to the large number of tested hypotheses.

## Results

The response rate was 72.1% (267 questionnaires were returned out of 370 distributed), which represented roughly the same percentage of all Slovenian intensivists. Termination of futile treatment was assessed as ethically acceptable (*P *0.001). The statement that there is no ethical distinction between withholding and withdrawing of treatment could not be confirmed (the answers 'there is a difference' and 'undecided' were less frequent, but not statistically significant; *P *= 0.216). A do-not-resuscitate order (DNR) was used more often than other withholding treatment limitations (*P *0.001). A DNR was used most frequently in internal medicine ICUs (*P *0.001; compared with paediatric and surgical ICUs). Withdrawal of inotropes or antibiotics was used more often than withdrawal of mechanical ventilation or extubation (66.7% vs. 12.0%; *P *0.001). Withdrawal of mechanical ventilation or extubation was more often used in the paediatric ICUs (21.7%) as compared with the internal medicine ICUs (19.6%) and the surgical ICUs (3%) (*P *0.001). Over two-thirds (70.6%) of intensivists were against termination of hydration, which would be more often used in the internal medicine ICUs (*P *0.001). Thirty-one percent of intensivists used written DNR orders.

## Conclusion

Termination of futile treatment was found to be ethically acceptable for Slovenian intensivists, although they were not convinced that withholding and withdrawing of treatment were ethically equal. A DNR would be used most often. Withdrawal of inotropes or antibiotics would be used more often than withdrawal of mechanical ventilation or extubation. Termination of artificial hydration would be rarely used in practice.

